# The global transcriptome of *Plasmodium falciparum* mid-stage gametocytes (stages II–IV) appears largely conserved and gametocyte-specific gene expression patterns vary in clinical isolates

**DOI:** 10.1128/spectrum.03820-22

**Published:** 2023-09-12

**Authors:** Jonas A. Kengne-Ouafo, Saikou Y. Bah, Alison Kemp, Lindsay Stewart, Lucas Amenga-Etego, Kirk W. Deitsch, Julian C. Rayner, Oliver Billker, Fred N. Binka, Colin J. Sutherland, Gordon A. Awandare, Britta C. Urban, Bismarck Dinko

**Affiliations:** 1 Department of Biochemistry, Cell and Molecular Biology, West African Centre for Cell Biology of Infectious Pathogens, University of Ghana, Accra, Ghana; 2 Vaccine and Immunity Theme, MRC Unit The Gambia at London School of Hygiene & Tropical Medicine, Banjul, Gambia; 3 Malaria Programme, Wellcome Sanger Institute, Wellcome Genome Campus, Cambridge, United Kingdom; 4 Department of Infection Biology, Faculty of Infectious and Tropical Diseases, London School of Hygiene and Tropical Medicine, London, United Kingdom; 5 Department of Microbiology and Immunology, Weill Medical College of Cornell University, New York City, New York, USA; 6 Cambridge Institute for Medical Research, University of Cambridge, Cambridge, United Kingdom; 7 Laboratory for Molecular Infection Medicine Sweden, Department of Molecular Biology, Umeå University, Umeå, Sweden; 8 Department of Epidemiology and Biostatistics, School of Public Health, University of Health and Allied Sciences, Ho, Ghana; 9 Faculty of Biological Sciences, Liverpool School of Tropical Medicine, Liverpool, United Kingdom; 10 Department of Biomedical Sciences, School of Basic and Biomedical Sciences, University of Health and Allied Sciences, Ho, Ghana; University of Huddersfield, Huddersfield, United Kingdom

**Keywords:** transcriptomics, *Plasmodium falciparum*, gametocytes, clinical isolates

## Abstract

Our overall understanding of the developmental biology of malaria parasites has been greatly enhanced by recent advances in transcriptomic analysis. However, most of these investigations rely on laboratory strains (LS) that were adapted into *in vitro* culture many years ago, and the transcriptomes of clinical isolates (CI) circulating in human populations have not been assessed. In this study, RNA-seq was used to compare the global transcriptome of mid-stage gametocytes derived from three short-term cultured CI, with gametocytes derived from the NF54 reference laboratory strain. The core transcriptome appeared to be consistent between CI- and LS-derived gametocyte preparations, but some important differences were also observed. A majority of gametocyte-specific genes (43/53) appear to have relatively higher expression in CI-derived gametocytes than in LS-derived gametocytes, but a K-means clustering analysis showed that genes involved in flagellum- and microtubule-based processes (movement/motility) were more abundant in both groups, albeit with some differences between them. In addition, gametocytes from one CI described as CI group II gametocytes (CI:GGII) showed gene expression variation in the form of reduced gametocyte-specific gene expression compared to the other two CI-derived gametocytes (CI gametocyte group I, CI:GGI), although the mixed developmental stages used in our study is a potential confounder, only partially mitigated by the inclusion of multiple replicates for each CI. Overall, our study suggests that there may be subtle differences in the gene expression profiles of mid-stage gametocytes from CI relative to the NF54 reference strain of *Plasmodium falciparum*. Thus, it is necessary to deploy gametocyte-producing clinical parasite isolates to fully understand the diversity of gene expression strategies that may occur during the sequestered development of parasite sexual stages.

**IMPORTANCE:**

Maturing gametocytes of *Plasmodium falciparum* are known to sequester away from peripheral circulation into the bone marrow until they are mature. Blocking gametocyte sequestration can prevent malaria transmission from humans to mosquitoes, but most studies aim to understand gametocyte development utilizing long-term adapted laboratory lines instead of clinical isolates. This is a particular issue for our understanding of the sexual stages, which are known to decrease rapidly during adaptation to long-term culture, meaning that many LS are unable to produce transmissible gametocytes. Using RNA-seq, we investigated the global transcriptome of mid-stage gametocytes derived from three clinical isolates and a reference strain (NF54). This identified important differences in gene expression profiles between immature gametocytes of CI and the NF54 reference strain of *P*. *falciparum*, suggesting increased investment in gametocytogenesis in clinical isolates. Our transcriptomic data highlight the use of clinical isolates in studying the morphological, cellular features and molecular biology of gametocytes.

## INTRODUCTION

Malaria remains a major global health burden claiming the lives of hundreds of thousands of individuals each year, primarily African children under the age of 5 years (1). Most of the cases and deaths are due to *Plasmodium falciparum* infections, with higher rates in rural and remote areas with limited access to adequate treatment (2). The intraerythrocytic stages of *P. falciparum* comprise both asexual cycle of parasite replication through a process referred to as schizogony and a non-replicative sexual development process that is essential for subsequent transmission to the anopheline mosquito. This latter phase is initiated by a small proportion of asexual parasites that commit to the formation of the sexual stages termed gametocytes which, at maturity, are taken up by mosquitoes during blood feeding, allowing subsequent intra-mosquito development and transmission to other humans. In contrast to other *Plasmodium* species, gametocytes of *P. falciparum* develop through four morphologically distinct stages while sequestered in tissues of the bone marrow (stages I–IV) (3 – 6), before emerging into the peripheral circulation when mature (stage V), where they can be ingested by mosquitoes. During gametocyte development, a poorly understood process, gametocyte-specific genes that govern sequestration, maintenance in sequestration sites, and subsequent release into circulation are expressed (3 – 5, 7 – 9). It is not yet known which exact parasite-specific molecules directly mediate gametocyte sequestration, although multiple candidates have been proposed and investigated (10 – 14). In addition, it has also been shown that alterations in membrane deformability of the host erythrocyte and infection of erythroblasts by gametocyte-committed trophozoites (rings) in the bone marrow can contribute to gametocyte sequestration (15, 16).

Sustained efforts have led to a 37% drop in global malaria disease incidence since 2000, although there are troubling signs that this downward trajectory is flattening if not reversing (1). Malaria control is further complicated by the emergence and spread of parasite resistance to frontline antimalarials, vector resistance to insecticides, and the continued lack of an efficacious vaccine (1). Identifying novel drug and vaccine targets requires knowledge of parasite biology across its complex life cycle, which has been greatly improved by the advent of transcriptomics. This allows a better understanding of stage-specific transcriptional regulation which, in turn, enlightens the molecular mechanisms governing parasite survival, development, sexual switching, pathogenesis, and transmission (17).

A suite of transcript analysis tools, collectively known as RNA-seq, is being increasingly used to improve *Plasmodium* transcriptome coverage and resolution, leading to the identification of new transcripts and alternative splicing events (18), the deciphering of signatures of sexual commitment (19), and the elucidation of gene regulation by antisense RNA (20). Among the RNA-seq analysis approaches available, a directional amplification-free transcriptome sequencing (DAFTseq) approach was recently developed, which eliminates the A-T biases that are generated during PCR amplification and random priming in more standard RNA-seq methods, allowing full-length strand-specific whole transcript sequencing (21 – 23).

It is important to note that previous transcriptomic studies have mostly evaluated *P. falciparum* reference laboratory-adapted strains (LS) (24). These strains have been grown in *in vitro* culture for long periods of time, in some cases having been adapted in the 1970s, and as such they may not reflect a true picture of contemporary parasite populations that are actively circulating in malaria-endemic regions. At the genomic level, LS also show evidence of polymorphisms, some of which introduce premature stop codons with detrimental effects on the structure and function of the corresponding proteins (25, 26), and sub-telomeric chromosome segment loss, gene deletions and insertions have also been characterized in LS which appear to be absent in clinical isolates (CI) (27 – 31). Some of these may have provided selective growth advantages specific for *in vitro* culture and that would be selected against natural conditions, for example, genes required for sexual commitment (26, 32).

Despite the fact that LS appear to be distinct from their CI counterparts, parasite culture adaptation to growth *in vitro* remains a gold standard method as it allows experimental investigation of all developmental stages in synchronized preparations of *P. falciparum* with a high level of purity. By contrast, clinical samples collected directly from patients do not include mature trophozoites or schizonts from the asexual cycle or mid-stage gametocytes from the sexual stages as these are sequestered away from the peripheral circulation and can also contain multiple different *P. falciparum* strains, a feature referred to as mixed infections (3 – 5, 7 – 9). Recently, culture-adapted CI samples are potentially useful in allowing access to all parasite stages while also mitigating the risk of large-scale genomic and transcriptomic changes acquired by LS during long-term culture adaptation. However, there are limited data comparing the transcriptional profiles between asexual and sexual stage parasites of CI of *P. falciparum,* and none at all investigating gene expression patterns of mid-stage gametocytes derived from recently adapted CI (20, 24).

In this study, we report RNA-seq data for mid-stage gametocytes and their asexual progenitors (trophozoites and schizonts) generated from short-term cultured CI and one reference LS line. Using the DAFT-seq approach, we found that the transcriptome of gametocytes derived from recent clinical isolates of *P. falciparum* appears quantitatively and qualitatively distinct from each other, and the transcriptome of gametocytes derived from NF54 cultures. Studies geared toward drug and vaccine development against sexual stages of the malaria parasite will benefit from a better understanding of sexual stage transcriptomes in CI-derived developmental stages.

## RESULTS

### Transcriptome of midstage gametocytes and trophozoites/schizonts

**Fig 1:**
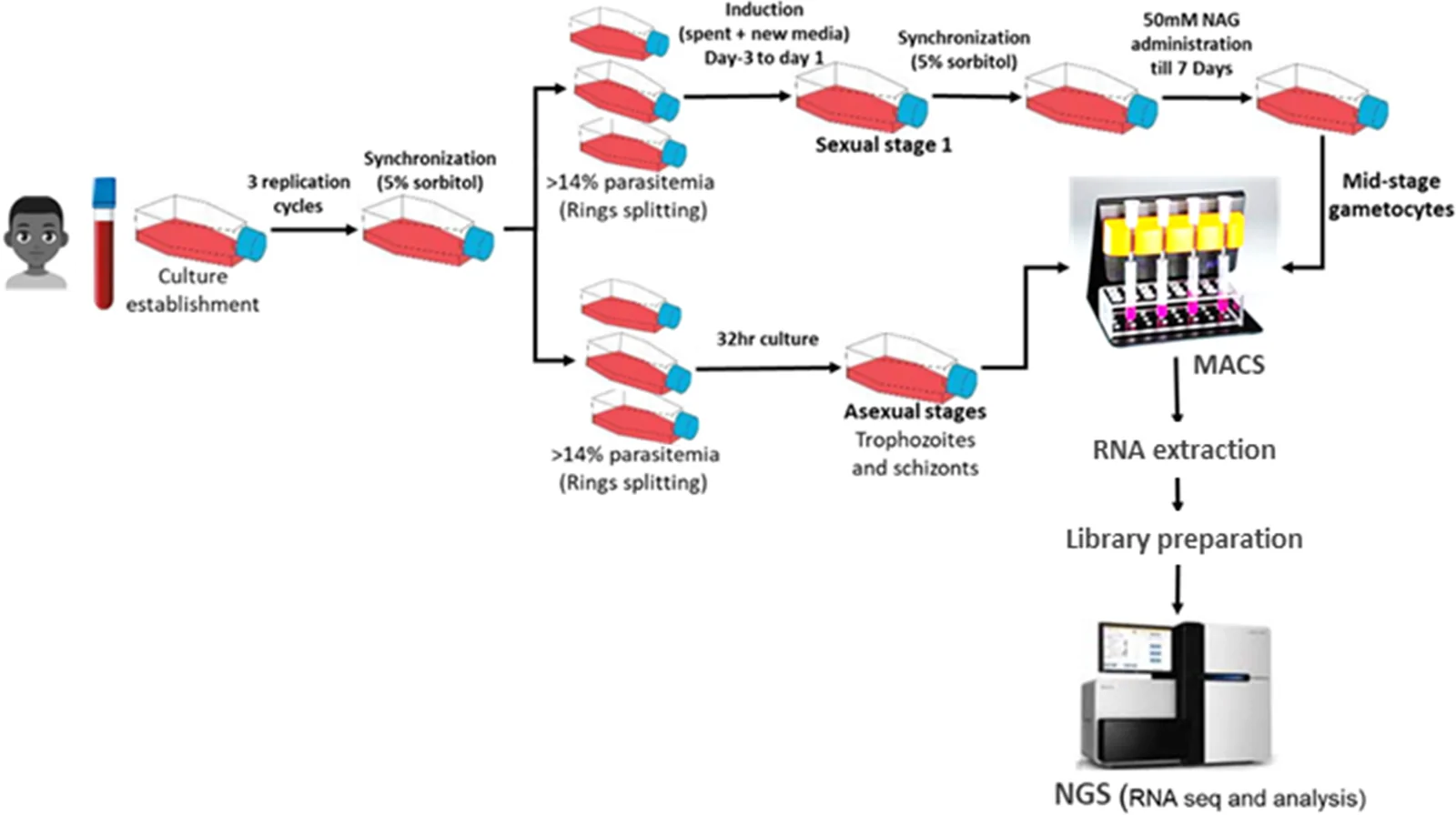
Experimental design for RNAseq of maturing gametocytes derived from clinical isolates and laboratory reference strain NF54 and their asexual progenitors.

To investigate the patterns of gene expression in stages II–IV of developing gametocyte development, which cannot be isolated from peripheral blood, mixed preparations of gametocyte-infected red blood cells (stages II–IV) were derived from CI HL1212 (33), Gh285, and Gh282, with laboratory strain (LS) comparator NF54, and subjected to DAFT-seq analysis. Two or three biological replicates were analyzed for each CI and NF54 (Fig. 1; Tables 1 and 2; Table S1). Transcriptomes were also generated from asexual progenitors of each preparation for all four parasite lines.

**TABLE 1:** Stage composition and yield of 10 RNA preparations from mixed mid-stage gametocyte cultures after magnetic-activated cell sorting (MACS)

Replicate	Parasite strain	II (%)	III (%)	IV (%)	Total gametocytemia/200 RBCs (%)^*a*^	Neubauer count per mL after MACS (10^6^)	Total in 2 mL (10^6^)^*b*^	RNA µg/µL	Total µg/45 µL
1	HL1212	2 (15)	3 (23)	8 (34)	13 (6.5)	3.10	6.20	0.19	8.73
	Gh282	3 (21)	4 (29)	7 (35)	14 (7)	3.60	7.20	0.06	2.89
	Gh285	3 (21)	5 (36)	6 (37)	14 (7)	2.50	5.00	0.05	2.43
	NF54	4 (25)	7 (38)	5 (31)	16 (8)	4.00	8.00	0.05	2.43
2	HL1212	3 (21)	4 (29)	7 (35)	14 (7)	2.80	5.60	0.08	3.55
	Gh282	3 (24)	5 (39)	5 (38)	13 (6.5)	4.10	8.20	0.02	1.09
	Gh285	2 (18)	4 (36)	5 (40)	11 (5.5)	3.30	6.60	0.04	1.62
	NF54	4 (24)	5 (29)	8 (41)	17 (8.5)	3.60	7.20	0.42	18.86
3	HL1212	3 (21)	5 (36)	6 (37)	14 (7)	2.80	5.60	0.02	0.68
	Gh282								
	Gh285								
	NF54	3 (21)	6 (37)	5 (36)	14 (7)	3.50	7.00	0.19	8.37

^*a*^ Gametocyte counts were made after pooling four 50 mL cultures (approximately 1–1.5mL packed RBCs per flask), and a slide was stained with Giemsa.

^*b*^ In the case of percentage gametocytemia, an average of two counts is represented, each against 200 RBC. In the case of the Neubauer estimated gametocyte count of absolute numbers, a 1/20 dilution of the MACS-purified gametocytes was made from the 2 mL total volume to count and to determine the gametocytes per microliter and the total number of gametocytes/2 mL estimated.

**TABLE 2:** Stage composition and estimated yield of 12 asexual parasite stage RNA preparations after MACS purification

Replicate	Parasite strain	Trophozoites (%)	Schizonts (%)	Total/200 RBCs (%)	Neubauer count per mL after MACS (10^6^)^*a*^	Total in 2 mL (10^6^)^*b*^	RNA µg/µL	Total RNA µg/45 µL
1	HL1212	8 (42)	12 (60)	20 (10)	5.2	10.4	0.42	18.86
	Gh282	10 (53)	9 (41)	19 (9.5)	4.8	9.6	0.14	6.30
	Gh285	7 (43)	11 (61)	18 (9)	4.4	8.8	0.54	24.29
	NF54	12 (46)	14 (54)	26 (13)	6.4	12.8	1.82	81.85
2	HL1212	11 (58)	8 (44)	19 (9.5)	4.6	9.2	1.52	68.39
	Gh282	13 (54)	11 (46)	24 (12)	5.7	11.4	0.36	16.40
	Gh285	12 (57)	9 (37)	21 (10.5)	5.3	10.6	0.14	6.35
	NF54	10 (59)	7 (45)	17 (8.5)	4.2	8.4	0.17	7.70
3	HL1212	12 (63)	7 (46)	19 (9.5)	4.4	8.8	0.09	4.05
	Gh282	10 (56)	8 (38)	18 (9)	4.1	8.2	0.45	20.16
	Gh285	4 (33)	8 (47)	12 (6)	2.2	4.4	0.12	5.40
	NF54	13 (52)	12 (48)	25 (12.5)	6.1	12.2	5.82	262.09

^*a*^ Trophozoite/schizont counts were made after pooling four 50 mL cultures (approximately 1–1.5 mL packed RBCs per flask), and a slide was stained with Giemsa.

^*b*^ In the case of percentage parasitemia, an average of two counts are represented, each against 200 RBC. In the case of the Neubauer estimated parasite count of absolute numbers, a 1/20 dilution of the MACS-purified asexual parasites was made from the 2 mL total volume to count and to determine the parasites per microliter and the total number of asexual parasites/2 mL estimated.

**Fig 2:**
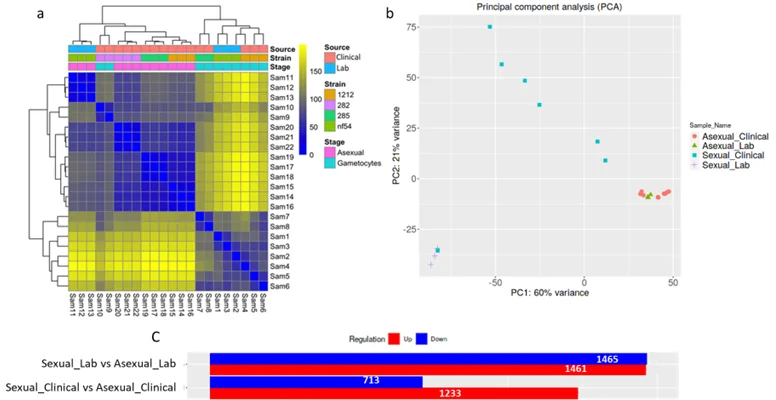
Using RNAseq to identify similarities and differences between strains. (a) Heatmap generated based on distance matrix comparing total gene expression within and between samples, (b) principal component analysis plot showing clustering of different developmental stages of CI and NF54 laboratory strain (LS), *P. falciparum* based on total gene expression. (c) A number of differentially expressed genes for CI and LS.

After quality control of the raw data, we performed read-trimming, mapping, transcript quantification, and data transformation (Fig. S2) and generated the sample correlation heatmap by computing a distance matrix between samples. After mapping, more than 90% of the reads from all the samples mapped to the 3D7 reference genome with LS having more properly paired reads (>90%) than CI (70–90%) (Table S2). As shown in Fig. 2a, samples mainly clustered hierarchically based on developmental stage rather than strain origin, except Gh282, whose gametocyte preparations in the analysis clustered with the asexual stages from other strains. A similar observation was made in principal component analysis (Fig. 2b), with clustering primarily based on stages and not strains, with the exception of the same two gametocyte samples from Gh282. For clarity in the rest of the analysis, these two data sets from Gh282 were together considered to comprise CI gametocyte group II (CI:GGII) as a distinct group, whereas the other five CI gametocyte preparations were considered to comprise CI gametocyte group I (CI:GGI). Unless otherwise stated, CI gametocytes will refer to CI gametocyte group I (CI:GGI). Globally, more genes appeared to be differentially expressed between sexual and asexual stages within the LS (2,926 genes) than within CI (1,946 genes), and in *CIs*, more genes were upregulated in sexual stages than were downregulated (Fig. 2c). Overall fewer genes were identified as expressed in the CI, but this may be partly due to mapping issues, as all transcriptomes were mapped against the published 3D7 reference genome, which is a derivative of NF54 (the LS used in this analysis) and so sequencing data from NF54 is expected to map to the reference much more readily.

### Comparison of stage-specific transcriptomes within *P. falciparum* clinical isolates and laboratory reference

**Fig 3:**
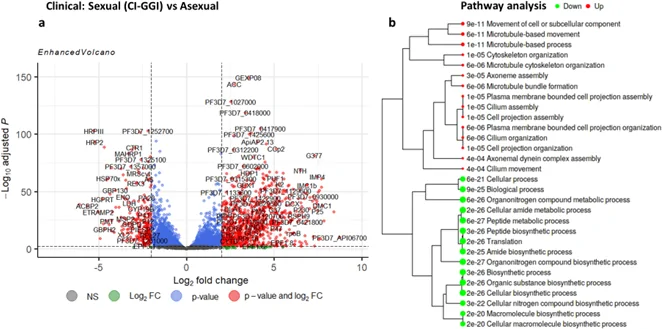
Comparison between clinical isolate gametocyte group I (CI:GGI) and asexual stages. (a) Volcano plot showing genes that are differentially expressed between sexual and asexual parasites of CI parasites (log2 fold change [FC] threshold set at 2 and the *P* value at 0.05). (b) Pathway analysis, dendrogram showing biological processes that are affected by the differentially expressed genes. The diameter of the circle on the dendrogram reflects the *P* value. The lower the *P* value, the bigger the diameter.

**Fig 4:**
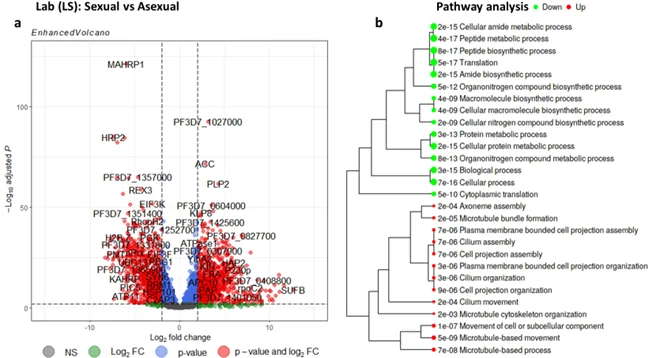
Comparison between LS-derived gametocyte and asexual stages. (a) Volcano plot showing genes that are differentially expressed between sexual and asexual parasites of NF54 LS (log2 fold change [FC] threshold set at 2 and the *P* value at 0.05). (b) Pathway analysis, dendrogram showing biological processes that are affected by the differentially expressed genes. The diameter of the circle on the dendrogram reflects the *P* value. The lower the *P* value, the bigger the diameter.

**Fig 5:**
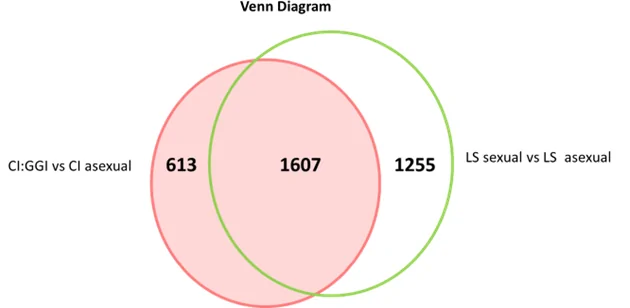
Venn diagram showing numbers of differentially expressed genes in both clinical isolates and the laboratory strain NF54 parasites upon comparing sexual against asexual stages (analysis done only with CI:GGI sexual stages).

A total of 2,220 (881 downregulated and 1,339 upregulated) genes were found to be differentially expressed between the sexual (CI:GGI) and asexual stages of CI while 2,862 (1,453 downregulated and 1,409 upregulated) genes were differentially expressed between those of the LS (Fig. 3a and 4a). In all, 1,607 genes were differentially expressed in sexual stages in both clinical isolates and the laboratory strain, meaning the majority of the sexual stage transcriptome is conserved between CI and LS. However, 613 and 1,255 genes were differentially expressed only in CI and LS parasites, respectively (Fig. 5). Genes commonly upregulated in both CI and LS gametocytes included putative secreted ookinete protein, ookinete surface protein P25, gametocyte-specific protein, 6-cysteine protein P230, male development gene 1, 6-cysteine protein P47, oocyst capsule protein Cap380, CCR4-NOT transcription complex subunit 2, and some AP2 domain transcription factors; many of these are well-known gametocyte markers (Fig. 3a and 4a; Table 3). However, while there were many similarities in the transcriptome of CI and LS, the majority of the gametocyte-specific genes showed higher fold changes in CI-derived gametocytes. Only 10 out of 53 gametocyte-specific genes in Table 3 had relatively higher expression in LS-derived gametocytes, including the male gamete fusion factor HAP2, oocyst capsule protein Cap380, 6-cysteine protein P230p, sporozoite invasion-associated proteins, and a putative AP2 domain transcription factor (PF3D7_1305200) (Table 3). However, some genes were found to be downregulated or not expressed at all in LS-derived gametocytes while being upregulated in CI gametocytes (CI:GGI). These include the 6-cysteine protein (P48/45), gamete antigen 27/25, gametocyte-exported protein 2, putative AP-2 complex subunit sigma, a member of the Plasmodium-exported protein (PHISTc) family of unknown function, and a flagellar outer arm dynein-associated protein (Table 3; Tables S3-1 and S3-2)

**TABLE 3:** Gametocyte-specific genes differentially expressed between sexual and asexual parasites in both clinical isolates and laboratory reference strain

No	Gene ID	Gene name	Product description	Log2 fold change (CI : NF54)	Adj. *P* value (CI)	Adj. *P* value (NF54)
1	PF3D7_0518800	PSOP13	Secreted ookinete protein, putative	(8.4 : 2.2)	6.02E−33	4.16E−02
2	PF3D7_1031000	P25	Ookinete surface protein P25	(7.5 : 1.6)	1.26E−33	1.01E−01^*a*^
3	PF3D7_1038400	Pf11-1	Gametocyte-specific protein	(7.3 : 7.5)	7.94E−44	6.86E−22
4	PF3D7_0209000	P230	6-Cysteine protein P230	(6.6 : 3.8)	6.81E−35	1.79E−06
5	PF3D7_0630200	PSOP6	Secreted ookinete protein, putative	(5.9 : 2.8)	1.28E−24	1.13E−03
6	PF3D7_1216500	MDV1	Male development gene 1	(5.3 : 2.7)	4.14E−17	5.93E−03
7	PF3D7_1346800	P47	6-Cysteine protein P47	(5.1 : 2.2)	2.45E−18	1.61E−02
8	PF3D7_0320400	Cap380	Oocyst capsule protein Cap380	(5.1 : 6.4)	1.48E−24	5.04E−18
9	PF3D7_1128600	NOT2	CCR4-NOT transcription complex subunit 2, putative	(5.0 : 1.9)	2.28E−32	3.31E−03
10	PF3D7_1346700	P48/45	6-Cysteine protein	(4.6 : −0.1)	1.23E−21	9.28E−01^*a*^
11	PF3D7_1429200	ApiAP2	AP2 domain transcription factor AP2-O3, putative	(4.6 : 3.8)	2.01E−41	3.59E−14
12	PF3D7_1014200	HAP2	Male gamete fusion factor HAP2, putative	(4.5 : 6.0)	3.62E−28	2.47E−23
13	PF3D7_0721700	PSOP1	Secreted ookinete protein, putative	(4.4 : 3.1)	1.50E−36	2.81E−09
14	PF3D7_0831300	GEXP13	Plasmodium-exported protein, unknown function	(4.4 : 0.9)	5.03E−30	1.44E−01^*a*^
15	PF3D7_1020100	N/A	Flagellar outer arm dynein-associated protein, putative	(4.3 : 0.6)	9.82E−12	5.60E−01^*a*^
16	PF3D7_0208900	P230p	6-Cysteine protein P230p	(4.2 : 6.5)	4.02E−18	1.36E−19
17	PF3D7_1350900	ApiAP2	AP2 domain transcription factor AP2-O4, putative	(4.1 : 2.4)	4.14E−94	1.06E−15
18	PF3D7_1234400	MiGS	Microgamete surface protein MiGS, putative	(4 : 4.9)	3.02E−23	3.23E−16
19	PF3D7_1218800	PSOP17	Secreted ookinete protein, putative	(3.9 : 1.1)	1.46E−37	1.73E−02
20	PF3D7_1449000	GEST	Gamete egress and sporozoite traversal protein, putative	(3.8 : 2.8)	1.16E−14	1.65E−04
21	PF3D7_0515600	GEP	Gamete egress protein GEP, putative	(3.7 : 2.8)	1.95E−60	5.85E−17
22	PF3D7_0731800	GEXP08	Alpha/beta hydrolase, putative	(3.5 : 1.5)	2.87E−150	3.47E−13
23	PF3D7_1372100	GEXP04	Plasmodium-exported protein (PHISTb), unknown function	(3.4 : −0.1)	5.75E−15	9.03E−01^*a*^
24	PF3D7_0513700	PSOP12	Secreted ookinete protein, putative	(3.2 : 3.8)	5.41E−21	1.20E−13
25	PF3D7_1216600	CelTOS	Cell traversal protein for ookinetes and sporozoites	(3.2 : 4.5)	2.67E−08	1.19E−07
26	PF3D7_0515500	GEP1	Gametogenesis essential protein 1, putative	(3.1 : 2.2)	1.45E−48	5.13E−13
27	PF3D7_0404100	ApiAP2	AP2 domain transcription factor AP2-SP2, putative	(3.1 : 3)	7.95E−14	9.23E−07
28	PF3D7_1340000	PSOP7	Secreted ookinete protein, putative	(2.7 : 3.9)	4.68E−24	6.70E−23
29	PF3D7_0830300	SIAP2	Sporozoite invasion-associated protein 2	(2.6 : 4.3)	4.59E−10	3.02E−12
30	PF3D7_0936600	GEXP5	Gametocyte-exported protein 5	(2.5 : 2.5)	6.18E−08	3.17E−04
31	PF3D7_0812300	subsp.	Sporozoite surface protein 3, putative	(2.4 : 3)	2.80E−11	1.47E−08
32	PF3D7_0408600	SIAP1	Sporozoite invasion-associated protein 1	(2.2 : 4)	3.03E−07	3.90E−10
33	PF3D7_1302100	G27/25	Gamete antigen 27/25	(2.2 : −4.8)	2.51E−04	2.54E−08
34	PF3D7_1342900	AP2-HS	AP2 domain transcription factor AP2-HS	(2.1 : 1.3)	1.56E−41	6.30E−08
35	PF3D7_1143100	AP2-O	AP2 domain transcription factor AP2-O	(2 : 0)	1.03E−05	9.76E−01^*a*^
36	PF3D7_1317200	ApiAP2	AP2 domain transcription factor AP2-FG, putative	(2 : 2.2)	8.30E−84	1.69E−46
37	PF3D7_0715400	PSOP20	Secreted ookinete protein, putative	(1.8 : 0.2)	8.42E−05	7.93E−01^*a*^
38	PF3D7_1102500	GEXP02	Gametocyte exported protein 2	(1.7 : −1)	3.02E−07	5.53E−02^*a*^
39	PF3D7_1006100	NOT5	CCR4-NOT transcription complex subunit 5, putative	(1.7 : −1.5)	1.36E−06	5.24E−03
40	PF3D7_1305200	ApiAP2	AP2 domain transcription factor, putative	(1.3 : 2.1)	3.31E−08	1.82E−09
41	PF3D7_0404800	N/A	Sporozoite-specific protein S10, putative	(1.2 : 2.4)	3.51E−03	5.77E−05
42	PF3D7_1148700	GEXP12	Plasmodium-exported protein (PHISTc), unknown function	(1.1 : −4)	2.99E−05	9.78E−27
43	PF3D7_1342500	SPECT1	Sporozoite protein essential for cell traversal	(0.8 : 1.8)	6.32E−02^a^	2.68E−03
44	PF3D7_0217300	AP2S	AP-2 complex subunit sigma, putative	(0.1 : −5.3)	6.84E−01^a^	1.10E−31
45	PF3D7_0508000	P38	6-Cysteine protein	(−0.4 : −2.3)	3.02E−01^a^	1.08E−04
46	PF3D7_0805200	GAMER	Gamete release protein, putative	(−0.8 : −5)	1.96E−01^a^	4.21E−08
47	PF3D7_0935600	GIG	Gametocytogenesis-implicated protein	(−1 : −2.9)	5.42E−03	2.29E−09
48	PF3D7_0210000	Sec61-gamma	Secretory complex protein 61 gamma subunit	(−1.2 : −4.2)	2.48E−03	1.85E−12
49	PF3D7_1440700	AP3M	AP-3 complex subunit mu, putative	(−1.8 : −0.8)	3.99E−21	4.63E−03
50	PF3D7_1007700	AP2-I	AP2 domain transcription factor AP2-I	(−1.9 : −1.5)	2.58E−17	3.22E−06
51	PF3D7_0212600	SPATR	Secreted protein with altered thrombospondin repeat domain	(−1.9 : −1.5)	1.45E−05	2.31E−02
52	PF3D7_0612700	P12	6-Cysteine protein P12	(−2.2 : −2.1)	7.96E−25	2.97E−11
53	PF3D7_0404900	P41	6-Cysteine protein P41	(−2.4 : −1.9)	2.89E−30	1.18E−09

^*a*^ Not significant.

We also found many genes of unknown function among the top upregulated genes in gametocytes derived from clinical and laboratory strain parasites (Tables S3-1 and S3-2). For both LS and CI, genes coding DNA-/RNA-binding proteins, dynein heavy chains, and tubulin chains were differentially expressed between asexual and sexual stages in addition to conserved genes of unknown functions (Tables S3-1 and S3-2). A number of kinases and AP2 domain transcription factors (ApiAP2) were also observed to be differentially expressed in CI and LS gametocytes with relatively higher expression in CI-derived gametocytes (Table 4; Tables S3-1 and S3-2). The observations presented in this section were made upon comparing CI and LS gametocytes to their asexual counterparts, respectively, then the significant log2 fold changes (FC) were further compared between parasite strains.

**TABLE 4:** Selected kinases and AP2 domain transcription factors (ApiAP2) differentially expressed between sexual and asexual parasites in both clinical isolates and laboratory reference strain

No	Gene ID	Gene name	Product description	Log2 fold change (CI : NF54)	Adj. *P* value (CI)	Adj. *P* value (NF54)
1	PF3D7_1429200	ApiAP2	AP2 domain transcription factor AP2-O3, putative	(4.6 : 3.8)	2.01E−41	3.59E−14
2	PF3D7_1350900	ApiAP2	AP2 domain transcription factor AP2-O4, putative	(4.1 : 2.4)	4.14E−94	1.06E−15
3	PF3D7_0404100	ApiAP2	AP2 domain transcription factor AP2-SP2, putative	(3.1 : 3)	7.95E−14	0.000000923
4	PF3D7_0830300	SIAP2	Sporozoite invasion-associated protein 2	(2.6 : 4.3)	4.59E−10	3.02E−12
5	PF3D7_1342900	AP2-HS	AP2 domain transcription factor AP2-HS	(2.1 : 1.3)	1.56E−41	0.000000063
6	PF3D7_1143100	AP2-O	AP2 domain transcription factor AP2-O	(2 : 0.02)	0.0000103	0.975615677
7	PF3D7_1317200	ApiAP2	AP2 domain transcription factor AP2-FG, putative	(2 : 2.2)	8.3E−84	1.69E−46
8	PF3D7_1305200	ApiAP2	AP2 domain transcription factor, putative	(2.3 : 2.1)	3.31E−08	1.82E−09
10	PF3D7_1222600	AP2-G	AP2 domain transcription factor AP2-G	(1.1 : 2.3)	NA	NA
13	PF3D7_1107800	ApiAP2	AP2 domain transcription factor, putative	(0.2 : −1.8)	0.499112661	3.92E−09
14	PF3D7_0217300	AP2S	AP-2 complex subunit sigma, putative	(0.1 : −5.3)	0.684334371	1.1E−31
16	PF3D7_1115500	ApiAP2	AP2 domain transcription factor, putative	(−0.7 : −1.4)	0.004378104	0.0000829
18	PF3D7_1007700	AP2-I	AP2 domain transcription factor AP2-I	(−1.9 : −1.5)	2.58E−17	0.00000322
19	PF3D7_0320800	DOZI	ATP-dependent RNA helicase DDX6	(1.32: 0.71	3.67E−07	0.072052301
20	PF3D7_0525900	NEK2	NIMA-related kinase 2	(5.9 :3)	7.91E−26	0.000405092
21	PF3D7_1129600	N/A	Phosphatidylinositol-4-phosphate 5-kinase, putative	(5.6 :3.01)	2.59E−51	5.77E−08
22	PF3D7_0311400	PKRP	Kinase-related protein PKRP, putative	(5.1 :3.9)	3.04E−49	5.36E−14
23	PF3D7_0605600	N/A	Nucleoside diphosphate kinase, putative	(5.9 :5)	1.53E−25	1.74E−12
24	PF3D7_0816900	AK2	Adenylate kinase 2	(4.8 :3.5)	2.12E−56	1.60E−15
25	PF3D7_1113900	MAPK2	Mitogen-activated protein kinase 2	(4.4 :4.3)	1.59E−36	3.57E−17
26	PF3D7_0107600	eIK2	Eukaryotic translation initiation factor 2-alpha kinase 2, putative	(4.2 :3.7)	8.34E−53	2.76E−19
27	PF3D7_0719200	NEK4	NIMA-related kinase 4	(4.1 :2)	6.76E−27	0.000481319
28	PF3D7_1352600	N/A	Protein kinase, putative	(4 :2)	2.07E−70	1.91E−09
29	PF3D7_1201600	NEK3	NIMA-related kinase 3	(3.5 :2.1)	1.77E−26	2.73E−05
30	PF3D7_1454300	KIN	SNF1-related serine/threonine protein kinase KIN	(3 :3.2)	4.57E−42	1.04E−21
31	PF3D7_1219700	RKIP	raf kinase inhibitor	(3 :3.2)	3.33E−16	4.76E−09
32	PF3D7_0310100	CDPK3	Calcium-dependent protein kinase 3	(3 :4.6)	1.49E−12	1.47E−13
33	PF3D7_1315100	PK9	Serine/threonine protein kinase PK9	(2.9 :4)	1.93E−15	1.49E−13
34	PF3D7_1428500	N/A	Protein kinase, putative	(2.3 :1.8)	1.73E−66	1.01E−20
35	PF3D7_1246900	PKB	RAC-beta serine/threonine protein kinase	(2.2 :2)	4.00E−27	2.93E−10
36	PF3D7_0102600	FIKK1	Serine/threonine protein kinase, FIKK family	(1.7 :2.2)	0.000161208	0.000641146
37	PF3D7_0419900	N/A	Phosphatidylinositol 4-kinase, putative	(−0.4 :−2)	0.031346055	1.87E-14
38	PF3D7_1136500	CK1	Casein kinase 1	(−1 :−4.7)	0.003454426	3.59E-22
39	PF3D7_1223100	PKAr	cAMP-dependent protein kinase regulatory subunit	(−1.2 :−2.6)	4.83E−09	5.28E−18
40	PF3D7_1366500	NDK	Nucleoside diphosphate kinase	(−1.3 :−2.5)	1.49E−05	2.52E−08
41	PF3D7_1342400	CK2beta2	Casein kinase II beta chain	(−1.6 :−2)	7.03E−15	2.67E−11
42	PF3D7_1124600	EK	Ethanolamine kinase	(−2 :−1.7)	2.32E−21	5.04E−08
43	PF3D7_1108400	CK2alpha	Casein kinase 2, alpha subunit	(−2.2 :−3.2)	5.79E−22	1.19E−19
44	PF3D7_0934800	PKAc	cAMP-dependent protein kinase catalytic subunit	(−2.4 :−3.1)	5.23E−24	3.80E−19
45	PF3D7_1008900	AK1	Adenylate kinase	(−2.4 :−3.3)	3.84E−41	4.64E−35
46	PF3D7_0826700	RACK1	Receptor for activated c kinase	(−3 :−4.4)	1.08E−42	4.54E−42

Asexual stage-specific genes involved in RBC invasion such as RON4, ROP14, and RhopH2 (34, 35) were also significantly downregulated in sexual stages, as expected since gametocytes do not re-invade host cells as opposed to asexual stages (Fig. 3a and 4a). Pathway analysis of differentially expressed genes revealed microtubule-based and flagellum-based processes (movement and organization) to be significantly enriched in both CI:GGI and LS gametocytes. However, microtubule-based processes were significantly more enriched in CI:GGI parasites based on *P* values. Genes involved in metabolic processes were significantly depleted in gametocytes from both sources (Fig. 3b and 4b).

### Comparison between laboratory and clinical isolate transcriptomes reveals differential expression of gametocyte-specific and movement-related genes

**Fig 6:**
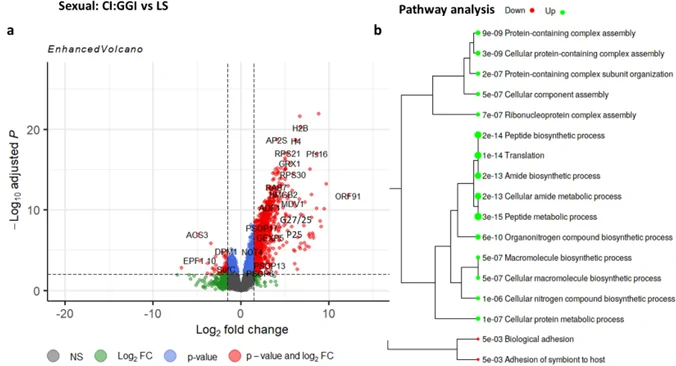
Comparison between CI- and LS-derived gametocytes. (a) Volcano plot showing genes that are differentially expressed between CI:GGI- and LS-derived gametocytes (log2FC threshold set at 2 and the *P* value at 0.05). (b) Pathway analysis, dendrogram showing biological processes that are affected by the differentially expressed genes. The diameter of the circle on the dendrogram reflects the *P* value. The lower the *P* value, the bigger the diameter.

Given that LS of *P. falciparum* are widely used as a reference for the study of parasite development, we compared expression profiles of LS and CI to determine whether they have similar developmental stage-specific transcriptomes. With respect to sexual stages, 657 genes (601 upregulated and 56 downregulated) were differentially expressed between gametocytes generated from CI and the NF54 LS at a log2FC of 2 and adj value of 0.05 (Fig. 6a; Table S3-3). Interestingly, these differentially expressed genes included some gametocyte-specific genes known to be involved in sexual stage parasite development, fertilization, and movement such as the male development gene 1, gamete antigen 27/25, ookinete surface protein P25, tubulin beta chain, putative dynein light chain, actins, and putative flagellar outer arm dynein-associated proteins (Table S3-3). Pathway analysis with differentially expressed genes showed mainly enrichment of biosynthetic processes (Fig. 6b).

**Fig 7:**
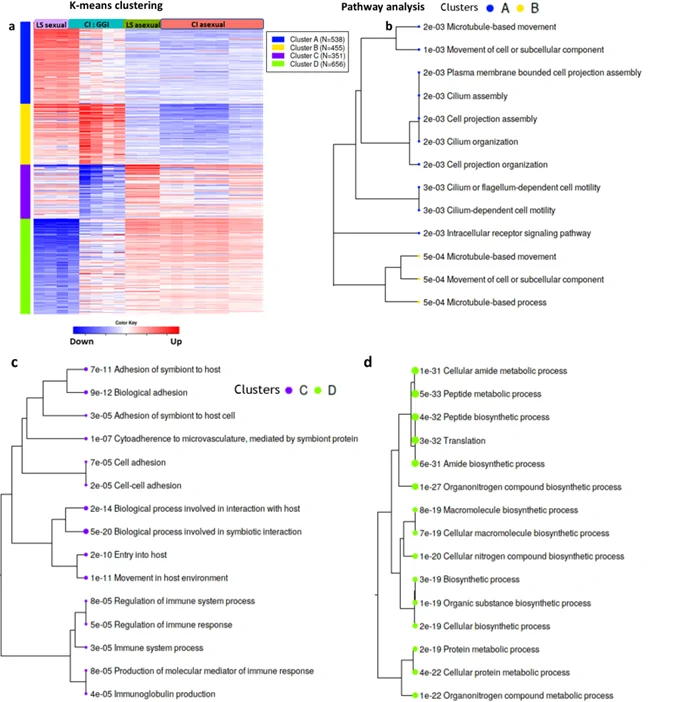
K-means clustering and pathway analysis. (a) Heatmap showing cluster A and cluster B genes upregulated in sexual stages of the parasites and clusters C and D upregulated in asexual stages of the parasites. (**b–**d) Pathway analysis with genes in clusters A–D. The diameter of the circle on the dendrogram reflects the *P* value. The lower the *P* value, the bigger the diameter. The analysis was done using the online platform for Integrated Differential Expression and Pathway analysis, idep95 (http://bioinformatics.sdstate.edu/idep95/).

To further delineate the differences between CI:GGI- and LS-derived gametocytes, we carried out K-means clustering analysis. This analysis revealed four gene clusters (A–D) among which clusters A and B were more expressed in LS- and CI-derived gametocytes, respectively, while clusters C and D were highly expressed in asexual stages irrespective of the parasite strain (Fig. 7a; Tables S3 and 4). Pathway analysis with cluster A and cluster B genes showed microtubule- and flagellum-based processes (movement/transport/motility) to be enriched. However, microtubule-based processes were significantly more enriched by cluster B than cluster A genes (Fig. 7b). Zooming into cluster A and cluster B genes, we found that they consisted mainly of cysteine repeat proteins, dyneins, tubulins, actins, and some conserved proteins of unknown function (Table 5). Apart from RSPH9 that was downregulated, all the genes in cluster B were found to be upregulated in CI:GGI gametocytes. These include among others: putative flagellar outer arm dynein-associated protein (PF3D7_1020100), alpha-tubulin 2, putative conserved proteins of unknown function (PF3D7_0828700 and PF3D7_1126700), and a putative GAS8-like protein (Table 5). These genes with exclusively high expression in CI:GGI parasites were involved in the regulation of flagellum movement, microtubule cytoskeleton organization, cell motility, and actin polymerization-dependent cell motility involved in migration within the mosquito host. On the other hand, all the genes in cluster A were upregulated in LS sexual parasites. Those genes were also found to be involved in microtubule cytoskeleton organization and cell motility (Table 5).

**TABLE 5:** Genes differentially expressed between CI:GGI and LS derived gametocytes following K-means clustering analysis

Gene ID	Gene name	Product description	Fold change	Adj. *P* value	GO term	GO ID
			(CI vs NF54)			
Cluster A						
PF3D7_0718300	CRMP2	Cysteine repeat modular protein 2	−4.9	0.074988	Intracellular receptor signaling pathway	GO:0030522
PF3D7_0616500	TLP	TRAP-like protein	−2.4	0.085502	Cell motility	GO:0048870
PF3D7_1465800	N/A	Dynein beta chain, putative	−3.5	0.002107	Microtubule-based movement	GO:0007018
PF3D7_1122900	N/A	Dynein heavy chain, putative	−2.5	0.002263	Microtubule-based movement	GO:0007018
PF3D7_1208200	CRMP3	Cysteine repeat modular protein 3	−2.4	0.002661	Intracellular receptor signaling pathway	GO:0030522
PF3D7_1104600	N/A	Radial spoke head protein, putative	−1.9	0.04922	Microtubule-based movement	GO:0003341
PF3D7_1475400	CRMP4	Cysteine repeat modular protein 4	−2.1	0.004381	Intracellular receptor signaling pathway	GO:0030522
PF3D7_1107600	N/A	Conserved Plasmodium protein, unknown function	−2.7	0.003758	Intracellular receptor signaling pathway	GO:0030522
PF3D7_0905600	WDR66	WD repeat-containing protein 66, putative	−3.0	0.004952	Motility	GO:0031514
PF3D7_0905300	N/A	Dynein heavy chain, putative	−2.8	0.004547	Outer dynein arm assembly	GO:0036158
PF3D7_1475700	N/A	Tubulin epsilon chain, putative	−2.7	0.007122	Microtubule cytoskeleton organization	GO:0000226
PF3D7_0508400	N/A	Conserved protein, unknown function	−3.4	0.00042	Positive regulation of cell motility	GO:2000147
PF3D7_0307300	EB1	Microtubule-associated protein RP/EB family, putative	−2.1	0.071596	Regulation of microtubule polymerization or depolymerization	GO:0031110
PF3D7_0933800	N/A	Tubulin delta chain, putative	−3.6	0.002895	Microtubule cytoskeleton organization	GO:0000226
PF3D7_1025500	N/A	Conserved Plasmodium protein, unknown function	−1.6	0.004376	Microtubule-based movement	GO:0007018
PF3D7_0818300	N/A	Dynactin subunit 6, putative	−1.9	0.021702	Mitotic spindle organization	GO:0007052
PF3D7_0922000	N/A	Dynein intermediate chain, putative	−1.4	0.072185	Movement	GO:0003341
PF3D7_1443600	N/A	Gamma-tubulin complex component, putative	−2.2	0.000926	Microtubule cytoskeleton organization	GO:0000226
PF3D7_0514000	TTL	Tubulin--tyrosine ligase, putative	−1.5	0.006797	Microtubule cytoskeleton organization	GO:0000226
Cluster B						
PF3D7_0517800	DCX	Apicortin	3.4	0.005172	Positive regulation of protein polymerization	GO:0032273
PF3D7_1215800	RSPH9	Radial spoke head protein 9, putative	−4.0	0.004369	Axoneme assembly	GO:0035082
PF3D7_1147200	N/A	Tubulin—tyrosine ligase, putative	2.8	0.046534	Microtubule cytoskeleton organization	GO:0000226
PF3D7_0724900	N/A	Kinesin-20, putative	2.1	0.232012	Microtubule-based movement	GO:0007018
PF3D7_1020300	N/A	Cytoplasmic dynein intermediate chain, putative	1.0	0.973733	Microtubule-based movement	GO:0007018
PF3D7_1020100	N/A	Flagellar outer arm dynein-associated protein, putative	4.2	0.018914	Microtubule-based movement	GO:0007018
PF3D7_0419000	N/A	Conserved Plasmodium protein, unknown function	2.1	0.010383	Regulation of cilium movement	GO:0003352
PF3D7_0422300	N/A	Alpha-tubulin 2	21.7	7.49E-12	Microtubule cytoskeleton organization	GO:0000226
PF3D7_0909500	SPM1	Subpellicular microtubule protein 1, putative	1.2	0.70902	Microtubule anchoring	GO:0034453
PF3D7_1118800	ARC40	Actin-related protein 2/3 complex subunit 1, putative	2.8	3.59E-05	Actin filament organization	GO:0007015
PF3D7_0828700	N/A	Conserved protein, unknown function	1.5	0.108154	Inner dynein arm assembly	GO:0036159
PF3D7_0624900	N/A	GAS8-like protein, putative	3.2	1.53E-05	Cell motility	GO:0048870
PF3D7_1412500	ACT2	Actin II	1.8	0.020577	Actin polymerization-dependent cell motility involved in migration within the mosquito host	GO:0070359
PF3D7_1126700	ATG23	Conserved Plasmodium protein, unknown function	3.3	0.00331	Cytoskeleton-dependent intracellular transport	GO:0030705

In comparison, transcripts for 141 genes were differentially expressed between asexual blood stages of clinical isolates and the NF54 laboratory reference parasites, of which 115 were less abundant and 26 more abundant, respectively, with the cut-off log2FC and adj. *P* value set at 2 and 0.05, respectively (Table S3-5). Genes for apical rhoptry neck protein, ring-exported proteins (REX), PhIL1 interacting protein PIP2 (PIP2), *Plasmodium*-exported proteins (PHISTs) of unknown function, exported protein families (EPF), non-coding RNAs, and some conserved *Plasmodium* genes with unknown function were found among the topmost differentially expressed genes between asexual stages of CI and LS. Most of these genes were less abundant in the CI asexual stages (Table S3-5). Of the 26 genes upregulated in CI, 10 were non-coding RNA alongside the 28S ribosomal RNA (Table S3-5). An MSP7-like protein (PF3D7_1334500) transcript was also abundant in CI implying an active mechanism of gene expression regulation specific to cell invasion-related genes (36).

Furthermore, we observed some background expression of gametocyte-specific genes, but this was more pronounced in the LS asexual stages compared to those of CI. These included 6-cysteine protein (P47), male development gene 1 (MDV1), putative gamete release protein (GAMER), and a putative secreted ookinete protein (PSOP13). Lastly, we observed a small number of transcripts that were more abundant in the asexual stages of the CI parasite lines, the majority of which were expressed by conserved genes with unknown functions (Table S3-5).

### Gametocytes derived from clinical isolates may be transcriptionally diverse

**Fig 8:**
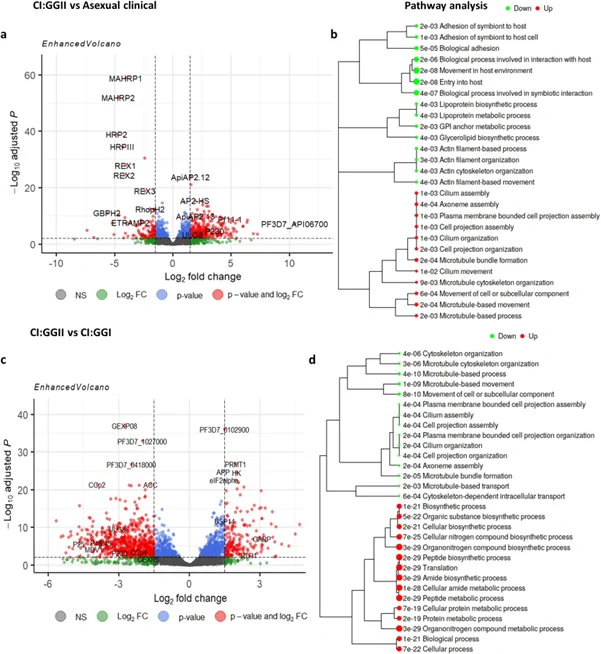
Differential gene expression analysis with CI:GGII parasites. (a) Volcano plot showing genes that are differentially expressed between clinical isolate gametocyte group II (outliers) and asexual stages of *CIs*. (b) Pathway analysis, dendrogram showing biological processes that are affected by the differentially expressed genes. (c) Volcano plot showing genes that are differentially expressed between clinical isolate gametocyte group II (outliers) and sexual stages of CI group I (log2FC threshold set at 2 and the *P* value at 0.05) (d) Pathway analysis, dendrogram showing biological processes that are affected by the differentially expressed genes. Log2FC threshold was set at 2 and the *P* value at 0.05. The diameter of the circle on the dendrogram reflects the *P* value. The lower the *P* value, the bigger the diameter.

Next, we performed a series of *in silico* tests to investigate what could explain the clustering of gametocyte transcription patterns from Gh282 (CI:GGII) with those of asexual stages. First, we compared the transcriptomes of these CI:GGII preparations to the transcriptome of CI asexual stages. As previously observed, this analysis revealed some gametocyte-specific genes among the top genes differentially expressed in CI:GGII and CI asexual parasites. These include 6-cysteine protein P230, AP2 domain transcription factors (ApiAP2), putative dynein heavy chain, putative nuclear formin-like protein MISFIT, (MISFIT), oocyst capsule protein Cap380, gametocyte-specific protein (Pf11-1), and NOT2 (Fig. 8a; Table S3-6). Pathway analysis demonstrated that the upregulated genes are involved in microtubule-based and flagellum-based processes (movement) while downregulated ones were found to be involved in host-parasite interaction processes (Fig. 8b). Combining CI:GGI and CI:GGII gametocytes and comparing them to their asexual stage progenitor led to a lower estimate of the number of differentially expressed genes from 2220 (CI:GGI only) to 1946 (CI:GGI and CI:GGII) (Fig. 2cand 3a).

Second, CI:GGII was compared to CI:GGI parasites. Transcripts from most gametocyte-specific genes, the secreted ookinete proteins and surface proteins (P25, PSOP13, SOAP, and PSOP17), male development gene 1 (MDV1), putative secreted ookinete protein (PSOP17), 6-cysteine proteins (P48/45, P230), putative flagellar outer arm dynein-associated protein (PF3D7_1020100), NOT2, GEXPO8, and 6-cysteine protein (P47) were found to be less abundant in the CI:GGII, suggesting lower expression of gametocyte-specific genes (Fig. 8c; Table S5-7). Transcripts that were more abundant in the CI:GGII transcriptomes were those encoding proteins involved in biosynthetic processes while the downregulated ones were involved in flagellum-based and microtubule-based processes (movement) as revealed by pathway analysis (Fig. 8d; Table S5-7). Similar results were obtained following a comparison of the CI:GGII transcriptome with that of sexual stages of NF54. Transcripts from gametocyte-specific genes were less abundant in the CI:GGII (Table S5-8).

Given that many of the differentially expressed genes were linked to flagellar function, genes that are specific to male gametocytes, we investigated whether the differences observed were not due to sex ratio fluctuations between strains. Specifically, the expression of female (CCp4) and male (PfMGET) gametocyte-specific genes was assessed as a proxy for female and male gametocyte abundance, respectively. Using normalized read, we compared the expression of these genes in different parasite groups (CI:GGI, CI:GGII, LS gametocytes, and their respective asexual stages). As expected, the expression level of the female-specific gene CCp4 was higher in CI:GGI and CI:GGII than that of the male-specific gene PfMGET (Fig S3a; Table 6). However, the expression of CCp4 was similar to that of PfMGET in LS gametocytes. We found no significant difference in the expression level of CCp4 between LS and CI:GGI parasites (Fig S3a; Table 6). On the other hand, the male-specific gene PfMGET was significantly more expressed in NF54 gametocytes compared to CI:GGI and CI:GGII (Fig S3a; Table 6). In asexual stages, the expression level of PfMGET was uniform across parasite strains (Fig S3b; Table 6). Thus, differences in sex ratios cannot account for the differences in gene expression that we observed between Gh282 and the other two CIs.

**TABLE 6:** Mean female (CCp4) and male (PfMGET) gametocyte-specific genes expression (normalized read counts)^*a*^

		Sexual parasites				Asexual parasites		
	Sexual_Clinical CI:GGI	Sexual_Clinical2 CI:GGII	Sexual_Lab	*P* value	Asexual_Clinical	Asexual_Clinical2	Asexual_Lab	*P* value
CCp4	14.2 ± 0.5	10.3 ± 2.3	13.8 ± 0.2	0.08	4.1 ± 0.2	7.2 ± 0.1	7.2 ± 0.6	0.01
PfMGET	10.2 ± 2.0	7.8 ± 0.3	13.9 ± 0.2	0.03	7.6 ± 0.9	6.8 ± 0.3	7.0 ± 0.1	0.01
*P* val	0.008	0.33	0.9		0.002	0.1	0.7	
F:M ratio	1.4	1.3	1.0		0.7	1.06	1.0	

^*a*^ Female and male gametocyte-specific gene expression was used as a proxy for the female:male ratio. Here normalized reads were compared between parasite groups in asexual and sexual parasites.

## DISCUSSION

In this study, we comprehensively compared the transcriptomes of mid-stage gametocytes from three different short-term adapted clinical African *P. falciparum* parasite lines with that of the NF54 reference line, using high-fidelity strand-specific RNA sequencing. We demonstrated that the core transcriptome of young gametocytes may be well conserved during the sexual development of short-term cultured clinical isolates (CI) and NF54, just as core transcriptomes have been found in previous studies that compared asexual stages of different *P. falciparum* LS (3D7, Dd2, and HB3) (22, 37). However, differences in transcript profiles were also found. We found multiple gametocyte-specific genes to be relatively more highly expressed in gametocytes derived from CI (CI:GGI) than NF54, and there were differentially expressed genes that were unique to either CI or LS. Strikingly, we also found some level of transcriptional diversity among the CI analyzed.

Our data provide the first evidence that transcriptional profiles of immature and mid-stage gametocytes, known to sequester in extravascular compartments of the bone marrow, of recently adapted CI strains may differ from the profiles seen in laboratory reference strains. This was marked by enhanced expression of some gametocyte-specific genes involved in gametocyte structural features (microtubules, actin, AP2 families, dyneins, crystalloid formation) in the CI, suggesting that studies of these cellular features may benefit from deploying recently adapted clinical parasite lines. It is also possible that the observed transcriptional diversity could be due in part to our use of mixed-stage developing gametocytes to generate the RNA-seq data sets, despite the fact that we deployed several replicates to minimize the effect of stage differences between samples. More tightly synchronized cultures and analysis of individual gametocyte stages, potentially utilizing single-cell technologies, would add extra information, but were simply impossible in the context of this study, where samples were collected in rural Ghana.

By analyzing transcriptomes arising from mid-stage gametocytes, comprising stages II–IV, which are known to sequester in the bone marrow and spleen, we found several gametocyte-specific gene transcripts to be highly expressed in gametocytes derived from clinical isolates. These included genes known to be involved in gametocyte commitment and subsequent development, fertilization of male and female gametes, as well as infectivity in the mosquito vector (36, 38 – 45). The enhanced expression of this group of genes was in contrast to the NF54 reference strain gametocyte transcriptome in which many gametocyte-specific genes showed lower expression. Vector transmission has been demonstrated to regulate *Plasmodium* parasite gene expression in the blood stages of rodent *Plasmodium* models (46), which could imply that recent host interaction has a role in maintaining sexual stage development gene expression in clinical isolates. It is also known that laboratory-adapted parasites such as 3D7, a clone of NF54, accrue chromosomal deletions and rearrangements over time (27, 28), which is probably related to a selection of variants that have more rapid *in vitro* growth. These genomic changes are expected to have a significant impact on parasite transcriptional profiles. To confirm that this observation was not due to differences in sex ratio between isolates, which is known to vary between parasite clones, the expression levels of CCp4 (female gametocyte specific) and PfMGET (male gametocyte specific) genes were assessed as a proxy for female/male gametocyte abundance. We found the expression level of CCp4 in LS and CI:GGI parasites to be similar. However, the expression level of PfMGET in LS gametocytes was far higher than that of CI:GGI parasites. We would have seen more expression of transmission and fertility-related genes in LS gametocytes instead if the sex ratio was a major confounder. This suggests that the differences observed are biological.

Additional upregulated genes in gametocytes of CI included kinases and AP2 domain transcription factor families (ApiAP2), which were also among the top differentially expressed genes in both CI and LS parasite lines, but exhibited much higher expression levels in the CI. The apiAP2 family of transcription factors comprises DNA-binding proteins that play a crucial role in developmental conversions throughout the *Plasmodium* life cycle (19, 47, 48). AP2-O transcripts were found exclusively upregulated in CI:GGI parasites. This gene is transcribed by intraerythrocytic female gametocytes but converted to proteins later during ookinete development in the mosquito. Its role is to induce the expression of genes involved in midgut invasion (49). DOZI protein was also upregulated in CI:GGI parasites. DOZI protein is known to inhibit the expression of midgut invasion-related genes including the transcription factor AP2-O, although their transcription is high in gametocytes (49). NF54 may have lost the expression of this gene due to its *in vitro* environment as it is rarely in contact with a mosquito’s midgut where invasion takes place. This could also explain the lack of expression of P48/45, G27/25, GEXP02, GEXP04, the putative AP-2 complex subunit sigma, and the putative flagellar outer arm dynein-associated protein in LS gametocytes, which are genes valuable for development and fertility of male gametes (49, 50) and erythrocyte cytoskeleton remodeling (51). This observation could be strain specific because P48/45 has been shown be to abundant in 3D7 stage II gametocytes although no comparison was done with CI gametocytes (20) and stage III in NF54 (41). In addition to P48/45, Van Biljon et al. also found *mdv1*, gamer, and Pfs16 to be highly expressed in NF54 gametocytes at day 6 in contrast to our findings. However, G27/25, PSOP12, PfS47, P230, and P25 were moderately expressed at day six in NF54 as observed in our study. Similar to our findings, data from Lopez-Barragan and colleagues (2011) in PlasmoDB also showed low expression of AP2-O, GEXP04, putative GAS8-like protein, and a conserved protein of unknown function (PF3D7_0828700) in 3D7 stage II gametocytes.

AP2-G is a member of the apiAP2 family that has been implicated in the regulation of sexual differentiation in *Plasmodium* (26, 32, 52). This gene showed no differential expression neither in CI nor in LS parasites. This corroborates findings by Van Biljon and colleagues who showed that NF54 gametocytes have mild expression of AP2-G on day 6 post-induction (41). This coincides with the day our gametocyte samples were harvested. This gene family is epigenetically controlled by reversible chromatin formation (in part) mediated by histone post-translational modifications. Epigenetic control is generally effected through phosphorylation, methylation, or acetylation of specific proteins such as histone residues mediated by *P. falciparum* heterochromatin protein 1 (PfHP1), histone deacetylase 2 (PfHda2), or methyltransferases of the SET family (19, 53 – 55). Such epigenetic mechanisms could explain the differential expression of specific kinases observed as well as the upregulated expression of the apiAP2 family members especially in *CIs*.

It is important to mention that we observed some background expression of gametocyte-specific genes more pronounced in the LS asexual stages compared to those of CI possibly indicating dysregulation of gene expression in the laboratory strains. It was also interesting to find some non-coding RNAs upregulated in CI asexual stage parasites suggesting active gene regulation in asexual *CIs* (36). These RNAs are known to play a prominent role in the regulation of gene expression and regulation of parasite virulence (56). On the other hand, some proportion (5%) of sexual stages contaminate the asexual NF54 preparation which may account for the upregulation of gametocyte-specific genes in the LS asexual transcriptome (57). However, we used stringent conditions in the production of gametocytes combining synchronization with 5% sorbitol, 50 mM GlcNAc treatment, and magnetic-activated cell sorting (MACS) purification in a bid to reduce the contamination rate in our samples. We also noticed a uniform low expression of the PfMGET gene in the asexual stages of CI and LS parasites implying a similarly low level of contamination in both CI and LS asexual stages by their sexual stage counterparts. This makes our observation more reliable.

Gametocytes of both CI and LS expressed a number of genes coding for DNA-/RNA-binding proteins which have been previously demonstrated to contribute to sexual commitment (32). Our data further show that transcripts encoding dynein heavy chains and tubulin subunits were differentially expressed in gametocytes of CI and LS. Dynein heavy chains and microtubules are important structural elements involved in locomotion, cell morphology, transport, and cell division (58, 59), and these were parasite cell activities highlighted in our gene ontology (GO) analysis. Both locomotion and cell division are key requirements for male gametocytes in particular (59, 60). K-means clustering analysis identified clusters A and B genes to be highly expressed in LS and CI gametocytes, respectively. Both clusters were made up of similar gene groups such as the actins, dyneins, tubulins, and flagellar proteins. Interestingly, the set of genes that were upregulated in CI gametocytes was different from the one in LS gametocytes, although both sets were shown through pathway analysis to be involved in flagellum-based or microtubule-based processes (movement, transport, and motility). It is likely that both parasite lines (CI and LS) may have adapted to preferentially express genes that help them thrive better in their respective environment. Moreover, some genes in cluster B were found to be highly expressed in CI:GGI gametocytes including those encoding the putative flagellar outer arm dynein-associated protein (PF3D7_1020100), alpha-tubulin 2, a conserved protein of unknown function (PF3D7_1126700), and a putative GAS8-like protein. As revealed by GO analysis, these genes have been implicated in the regulation of flagellum movement, microtubule cytoskeleton organization, cell motility, and actin polymerization-dependent cell motility involved in migration within the mosquito host. As such, they may play a significant role in disease transmission by *CIs*. Further characterization studies on the identified conserved proteins with the unknown function will be valuable as they could constitute potential transmission-blocking vaccine candidates. Worth noting is the presence of a putative kinesin-20 among cluster B genes in *CIs* (CI:GGI). This gene has been shown through single-cell RNA-Seq to be associated with mosquito stages and vector-to-host transmission (36).

Our principal component analysis revealed that both Gh282 sexual stage samples (CI:GGII) clustered together away from the majority of clinical isolate gametocyte (CI:GGI) transcriptomes and appeared closer to the asexual stage grouping, and displayed relatively low expression of a number of gametocyte-specific genes. Therefore, we performed a series of bioinformatic tests to eliminate the possibility of asexual stage contamination accounting for our observations. Our finding suggests that the CI:GGII were true gametocytes, and that these CI:GGII parasites are transcriptionally distinct from gametocytes of the other CI:GGI (25, 26, 37, 61). Further investigations utilizing gametocytes derived from additional CI may identify further examples of distinct transcriptional profiles, which may represent an adaptation to local ecological conditions and specific vectors. It is clearly risky to extrapolate from a single isolate, but it is worth noting this observation. This suggests that they may have different intrinsic transmissibility and could explain the variability observed in the infectivity index among malaria parasites in the natural environment which, in turn, influences disease transmission (62).

However, this study has a number of limitations. The low number of replicates of parasites that were used could have led to a less reliable conclusion. A previous study showed that 4–6 replicates gave more reliable results (24). Moreover, the transcriptome of gametocytes derived from CI was compared with only that of NF54 gametocytes, which may have a gene expression profile different from that of the other laboratory strains. We therefore recommend further studies with a larger number of isolates and replicates to complement the work described in this paper. Moreover, due to the limited amount of cDNA material, we could not validate our RNA-Seq results with quantitative PCR. However, we intensively compared our data with published findings and data available on PlasmoDB.

We also admit that it is naturally challenging to produce pure asexual stages of NF54 and CI when they are cultured at very high parasitemia, which is likely to have affected somehow the findings of this study. To minimize background gametocyte induction while maintaining parasites at parasitemias above 10%, culture media were changed daily or twice daily to reduce gametocyte commitment due to “stress.” Gametocyte induction by our protocol (63) follows the two-cycle gametocyte commitment model, where commitment can occur both during a single cycle level and also after an additional cycle of cell replication (64). NAG is applied to the cultures to stop asexual parasite growth, if any, on the fourth day after induction to allow both of these cycles to occur. To enhance gametocyte purity, we applied sorbitol on the first day of gametocytogenesis to eliminate the already dying trophozoites arising from uncommitted early trophozoites, leaving only pure stage I gametocytes, and these might be susceptible to sorbitol treatment due to the new permeation pathways, generated in gametocyte-infected erythrocytes (65). However, our gametocyte conversion output data suggest that this effect was minimal. Although not employed in this study, a single-cell RNA-seq approach would be ideal in overcoming these challenges and we recommend such a technology in future studies.

In this study, multigene families were excluded from the analysis because they are much more variable between clinical and laboratory isolates and many loci do not map directly to the 3D7 reference genome. Analysis of these genes requires long-read sequencing technology and *de novo* assembly; however, only short-read sequencing was done in our study. Future studies evaluating transcriptional differences in the multigene families between field and laboratory-adapted strains, which could lead to a better understanding of parasite biology, would require a different mix of sequencing technologies.

### Conclusion

Our understanding of *P. falciparum* gametocyte biology would be much improved if the mechanisms leading to gametocyte transmission were fully understood. Our work has highlighted the differential expression of gametocyte-specific and movement/motility-related genes important in gametocyte development, fertility, and transmission between gametocytes of clinical isolates and their laboratory comparators. Our preliminary evidence that different parasite lines may display distinct transcriptional programs in developing gametocytes further illustrates the importance of deploying recent clinical isolates in future studies especially those focusing on disease transmission.

## MATERIALS AND METHODS

### Clinical isolates and laboratory strain

*P. falciparum* Gh285, Gh282 *CIs*, laboratory line NF54 were obtained as low-passage liquid nitrogen frozen stocks from the laboratories of Prof. David Conway and Prof. David Baker at the London School of Hygiene and Tropical Medicine (LSHTM), UK. The HL1212 CI (no more than two expansions/passages from frozen stock since established) was isolated in 2012 and is of Nigerian origin (33). Isolates Gh285 and Gh282 (no more than two expansions/passages from frozen stock since established) were both collected from Navrongo, Ghana, in 2011 (66). All CI took 2–3 weeks to get established in culture after sampling from patients, meaning they had gone through a maximum of 10 intraerythrocytic cycles *in vitro* before experiments began. The use of the three *CIs* for the RNA sequencing analysis received ethical approval from the Research Ethics Committees of University College London Hospitals, UK. and the Ghana Health Service, Accra, Ghana. All methods of the study procedures were performed according to the Helsinki declarations.

### Gametocyte induction and MACS purification

Gametocytes were produced as described in references (10, 67 – 69) in O + blood using RPMI 1640 custom culture medium (Sigma) supplemented with 25 mM HEPES (Sigma), 10 mM D-glucose (Sigma), 50 mg/L hypoxanthine (Sigma), 10% AB human serum (obtained commercially), and gassed using a mix containing 3% CO_2_/1% O_2_/96% N_2_. Briefly, asexual blood stage parasites of 1.5 mL packed RBCs in 50 mL cultures kept at 2–4% hematocrit were allowed to pass through three cycles of replication before gametocyte induction. Before gametocyte induction, 5% sorbitol solution was used to synchronize the cultures during ring stage development. A tightly synchronized ring stage of >14% parasitemia was induced by the use of a proportionate amount of spent media, old media from previous asexual and gametocyte cultures, and an increase in hematocrit. Sorbitol solution (5%) was applied to the stage I gametocyte to eliminate trophozoites arising from non-committed ring stages and N-acetyl glucosamine, 50 mM (Sigma, UK), was applied throughout the period of gametocytocytogensis to kill any remaining asexual blood stages. Mid-stage gametocytes (containing a mixture of stages II–IV, as judged by microscopy) were harvested 5/6 d after induction.

Gametocytes were purified and enriched by magnetic separation using MACS columns (Miltenyi Biotech) based on the paramagnetic properties of gametocyte hemozoin as previously described (67). Eluted gametocytes were counted using a Neubauer cell counting chamber for counts per milliliter and the parasitemia confirmed by the Giemsa staining. As an extra precautionary step, purified parasites were incubated in 5% sorbitol for 5 min to kill any asexual parasites that might not have been visible by microscopy, thus predisposing asexual RNA to degradation.

Three independent gametocyte replicates for analysis were generated for NF54 and for each CI except CI Gh285 and Gh282, which had a very low gametocyte output in the third round. For comparison, asexual parasites of all four parasite lines comprising a mixture of trophozoites and schizonts were simultaneously harvested and taken through the same procedures as gametocytes.

### Sample collection and RNA isolation

Aliquots of replicate purified developing gametocytes and trophozoites/schizonts were lysed with 1× PBS/0.01% saponin and the parasite pellet was preserved in 1 mL Trizol after pooling similar aliquots together and stored at −80°C until RNA isolation.

RNA samples of developing gametocytes and trophozoites/schizonts were isolated using the phenol-chloroform method following an established protocol with little modifications (70). After isopropanol precipitation and washing with 75% ethanol, RNA samples were dissolved in 50 µL of diethylpyrocarbonate water, heated at 60–65°C for 5 min, and placed on ice. Then, aliquots of 5 µL were made in separately labeled tubes for bioanalysis to check RNA concentration and the remaining sets were stored at −80°C until use. Bioanalysis was done using the Agilent Bioanalyzer 6000 RNA Pico chip following the manufacturer’s instructions.

### RNA library preparation and directional amplification-free seq (DAFT-seq)

Library preparation and sequencing were carried out using the DAFT-seq protocol according to Chappel and colleagues (21) in the Sanger Institute pipeline for sequencing. Briefly, oligo-d(T) magnetic beads were used to pull out poly(A) mRNA molecules that were subsequently reverse-transcribed to cDNA using SuperScript II and oligo-d(T) as primers. Following second-strand synthesis, the double-stranded cDNA was broken down using a Covaris AFA sonicator. After fragmentation, dA-tailing, end repair, and adapter addition were performed following the established DAFT-seq protocol (22). cDNA libraries were then eluted in EB buffer and the second-strand cDNA was broken down using USER enzyme mix (NEB) to produce directional libraries that were finally quantified and subjected to sequencing on the Illumina HiSeq2000 (100 bp paired-end).

### Differential gene expression analysis

Generated reads were quality controlled using the FastQC Version 0.11.8 and trimmed using Version 0.38 of trimmomatic with the following settings (LEADING:3 TRAILING:3 SLIDINGWINDOW:4:15 MINLEN:36) to remove poor-quality reads and contaminating adapters. Cleaned reads were then mapped to Version 3 of the *P. falciparum* 3D7 reference genome (71) using the package HITSAT Version 2.2.0 and read counting was done using featureCount in the subread package Version 2.0.1 for differential expression analysis. Data normalization, clustering, and differential expression analysis were performed using Version1.28.1 of the DEseq2 package implemented in the R statistical environment in R studio software using default parameters. Prior to differential expression, principal component analysis and sample correlation were done to identify whether there were any stage-specific outliers. Differential expression analysis was done using the Wald test by default. Then, the outputs were visualized using the EnhancedVolcano package adjusting for multiple comparisons testing using the Benjamini and Hochberg method by default and setting a twofold change cutoff and *P* < 0.05. Multiple comparisons were made between asexual and sexual stages and between clinical isolates and the laboratory reference strain NF54.

### Pathway biology, gene enrichment, and K-means clustering analysis

Pathway biology and gene enrichment analysis were carried out to predict the biological processes or pathways that are likely to be enriched following differential expression of genes between the different comparison groups. K-means clustering analysis was performed to determine the genes that are more likely to be exclusively more expressed in clinical isolate-derived gametocytes and long-term adapted laboratory gametocytes. Both K-means clustering and pathway analyses were done using the online platform for Integrated Differential Expression and Pathway analysis, idep95 (http://bioinformatics.sdstate.edu/idep95/).

## Data Availability

The RNA sequencing data supporting the conclusions of this article are available in the Sequence Read Archive (SRA) of the National Center for Biotechnology Information (NCBI) of the National Institute of Health (NIH), USA, with accession number PRJNA736296. Additional resulting data sets are included in the article as well as the supplemental files.

## SUPPLEMENTAL MATERIAL

Figure S1 spectrum.03820-22-s0001.tif Giemsa parasite photos.

Figure S2 spectrum.03820-22-s0002.tif Read counts, data distribution, and density plots.

Figure S3 spectrum.03820-22-s0003.tif Sex ratio analysis.

Legends spectrum.03820-22-s0004.docx Legends for supplemental material.

Supplemental tables spectrum.03820-22-s0005.docx Tables S1 and S2.

Table S3 spectrum.03820-22-s0006.xlsx Differentially expressed genes for all the comparisons done.

ASM does not own the copyrights to Supplemental Material that may be linked to, or accessed through, an article. The authors have granted ASM a non-exclusive, world-wide license to publish the Supplemental Material files. Please contact the corresponding author directly for reuse.
