# Supplementary figures and images for: The global transcriptome of Plasmodium falciparum mid-stage gametocytes (stages II–IV) appears largely conserved and gametocyte-specific gene expression patterns vary in clinical isolates
Source: Microbiol Spectr. 2023 Sep 12;11(5):e03820-22. doi: 10.1128/spectrum.03820-22 (PMC10581088; doi:10.1128/spectrum.03820-22)

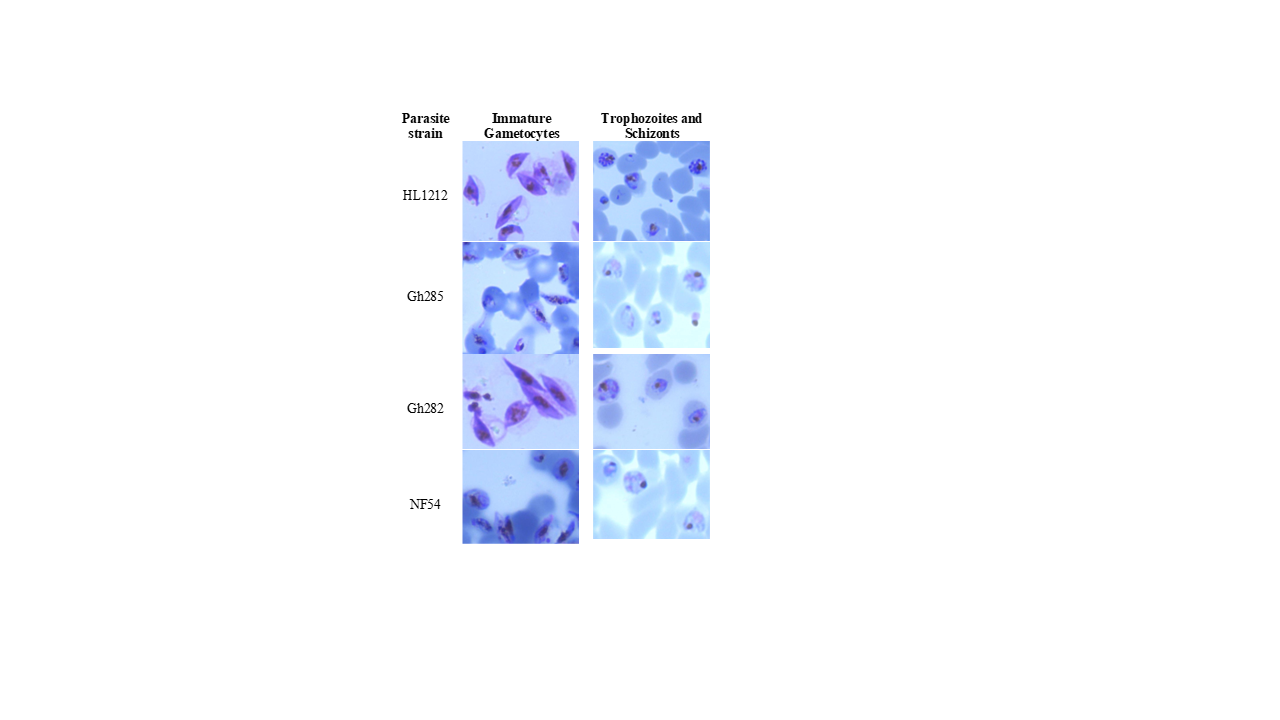

Supplement: Figure S1 — Giemsa parasite photos. [file spectrum.03820-22-s0001.tif]

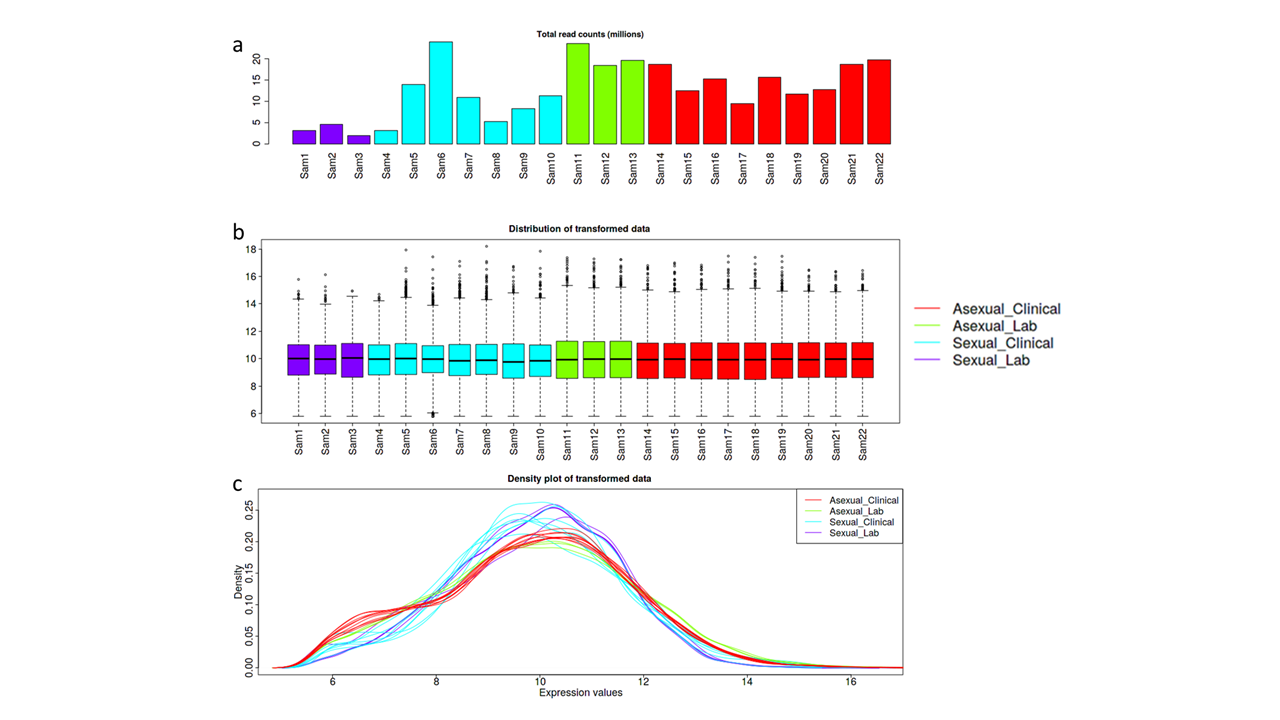

Supplement: Figure S2 — Read counts, data distribution, and density plots. [file spectrum.03820-22-s0002.tif]

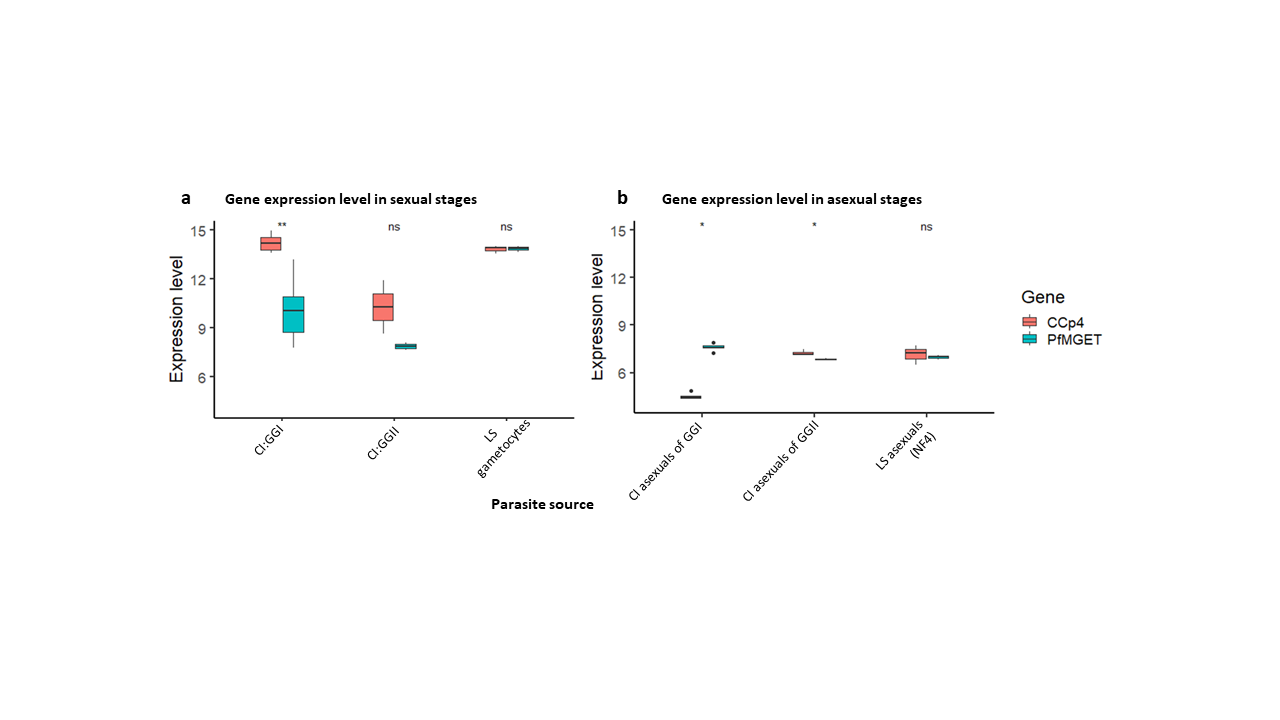

Supplement: Figure S3 — Sex ratio analysis. [file spectrum.03820-22-s0003.tif]
