# Supplementary material for: The global transcriptome of Plasmodium falciparum mid-stage gametocytes (stages II–IV) appears largely conserved and gametocyte-specific gene expression patterns vary in clinical isolates
Source: Microbiol Spectr. 2023 Sep 12;11(5):e03820-22. doi: 10.1128/spectrum.03820-22 (PMC10581088; doi:10.1128/spectrum.03820-22)
Supplement: Legends — for supplemental material. [file spectrum.03820-22-s0004.docx]

**Supplemental materials**

**Figures**

**Supplemental figure 1:** Representative sexual and asexual stages harvested for RNA-seq indicating the composition of mid-stage gametocytes and trophozoites/schizonts that were collected for the various parasite lines

**Supplemental Figure 2:** Data pre-processing, a) Library sizes per sample (total read counts), b) Distribution of transformed data, c) Density plot of transformed data. The transformation was done using Version1.28.1 of the DEseq2 package using the default normalization option implemented on the online platform for Integrated Differential Expression and Pathway analysis, idep95 (<http://bioinformatics.sdstate.edu/idep95/>).

**Supplemental Figure 3:** Female and male gametocyte-specific gene expression in clinical and lab parasites. a) Expression level of CCp4 (female gametocyte-specific) and PfMGET (male gametocyte-specific) in sexual stage parasites. b) Expression level of CCp4 (female gametocyte-specific) and PfMGET (male gametocyte-specific) in sexual stage parasites. The plot was drawn using normalized read counts.

**Tables**

**Table S1:** Number of asexual and sexual samples generated and used for RNAseq

**Table S2.** Bam files statistics for all the samples after mapping to the 3D7 reference genome

**Table S3.** Supplementary excel fine giving the differentially expressed genes for all the comparisons done
