# Supplementary material for: The global transcriptome of Plasmodium falciparum mid-stage gametocytes (stages II–IV) appears largely conserved and gametocyte-specific gene expression patterns vary in clinical isolates
Source: Microbiol Spectr. 2023 Sep 12;11(5):e03820-22. doi: 10.1128/spectrum.03820-22 (PMC10581088; doi:10.1128/spectrum.03820-22)
Supplement: Supplemental tables — Tables S1 and S2. [file spectrum.03820-22-s0005.docx]

**Table S1:** Number of asexual and sexual samples generated and used for RNA extraction

|  | Clinical isolates | | | Lab strain | |  | |  |
| --- | --- | --- | --- | --- | --- | --- | --- | --- |
|  | **HL1212** | **Gh282** | **Gh285** | | **NF54** | | Total | |
| Replicates (asexual) | 3 | 3 | 3 | | 3 | | 12 | |
| Replicates (sexual) | 3 | 2 | 2 | | 3 | | 10 | |
| **Total** | 6 | 5 | 5 | | 6 | | **22** | |

**Table S2:** Bam files statistics for all the samples after mapping to the 3D7 reference genome

| Sample ID | **Total reads** | **Reads mapped** | **Mapped (%)** | **Properly paired** | **Properly paired (%)** | **Singletons** | **Singletons (%)** | **Mapped to diff chrom** | **Mapped to diff chrom (mapQ>=5)** | **Stage** | **Source** |
| --- | --- | --- | --- | --- | --- | --- | --- | --- | --- | --- | --- |
| nf54 | 13855660 | 13147692 | 94.89 | 12070300 | 90.43 | 433578 | 3.25 | 60482 | 47706 | Sexual | Lab |
| nf54 | 15208998 | 14651224 | 96.33 | 13697840 | 93.03 | 369754 | 2.51 | 42978 | 35946 | Sexual | Lab |
| nf54 | 25758116 | 24648940 | 95.69 | 22685650 | 91.47 | 767102 | 3.09 | 119396 | 98296 | Sexual | Lab |
| 1212 | 14748411 | 13695677 | 92.86 | 11474750 | 86.82 | 567542 | 4.29 | 72142 | 57692 | Sexual | Clinical |
| 1212 | 16121618 | 15073137 | 93.5 | 13596590 | 87.04 | 771427 | 4.94 | 124508 | 102629 | Sexual | Clinical |
| 1212 | 28456432 | 26503231 | 93.14 | 23742248 | 86.69 | 1302811 | 4.76 | 230658 | 181907 | Sexual | Clinical |
| 285 | 12417949 | 11726429 | 94.43 | 10727630 | 88.92 | 512810 | 4.25 | 68822 | 55477 | Sexual | Clinical |
| 285 | 7946942 | 6200148 | 78.02 | 5261208 | 71.05 | 298608 | 4.03 | 58986 | 49294 | Sexual | Clinical |
| 282 | 13560293 | 12697300 | 93.64 | 9995950 | 88.38 | 370651 | 3.28 | 42218 | 31471 | Sexual | Clinical |
| 282 | 16945019 | 15700873 | 92.66 | 12693398 | 86.53 | 574556 | 3.92 | 97002 | 77522 | Sexual | Clinical |
| nf54 | 26772314 | 25890320 | 96.71 | 23973200 | 93.29 | 661372 | 2.57 | 85440 | 71353 | Asexual | Lab |
| nf54 | 20943928 | 20195884 | 96.43 | 18683896 | 92.85 | 544378 | 2.71 | 70510 | 59149 | Asexual | Lab |
| nf54 | 22243301 | 21502098 | 96.67 | 19946586 | 93.2 | 557409 | 2.6 | 70498 | 59678 | Asexual | Lab |
| 1212 | 21087930 | 19929073 | 94.5 | 18262998 | 89.39 | 825433 | 4.04 | 94586 | 76897 | Asexual | Clinical |
| 1212 | 14318074 | 13449568 | 93.93 | 12324262 | 88.86 | 562548 | 4.06 | 64768 | 51579 | Asexual | Clinical |
| 1212 | 17311412 | 16276745 | 94.02 | 14821886 | 88.42 | 771045 | 4.6 | 71302 | 58062 | Asexual | Clinical |
| 285 | 10795637 | 10240447 | 94.86 | 9381202 | 90.05 | 392824 | 3.77 | 45190 | 36435 | Asexual | Clinical |
| 285 | 17695246 | 16755936 | 94.69 | 15361492 | 89.73 | 673258 | 3.93 | 77434 | 63237 | Asexual | Clinical |
| 285 | 13337778 | 12577907 | 94.3 | 11519584 | 89.23 | 520133 | 4.03 | 58876 | 48417 | Asexual | Clinical |
| 282 | 14556432 | 13678227 | 93.97 | 12530020 | 88.91 | 577709 | 4.1 | 53670 | 44404 | Asexual | Clinical |
| 282 | 21242000 | 20041831 | 94.35 | 18353368 | 89.29 | 823689 | 4.01 | 93646 | 77620 | Asexual | Clinical |
| 282 | 22485062 | 21205415 | 94.31 | 19459636 | 89.36 | 854117 | 3.92 | 95478 | 78610 | Asexual | Clinical |

Diff chrom= different chromosome
